# Living arrangement of Indian elderly: a predominant predictor of their level of life satisfaction

**DOI:** 10.1186/s12877-023-03791-8

**Published:** 2023-02-10

**Authors:** Binayak Kandapan, Jalandhar Pradhan, Itishree Pradhan

**Affiliations:** Department of Humanities and Social Sciences, National Institute of Technology, Rourkela, Odisha 769008 India

**Keywords:** Life satisfaction, SWLS, Living Arrangement, Older Adults, LASI, India

## Abstract

**Objective:**

This article aims to examine the level of life satisfaction (LS) among Indian older adults and to determine whether their living arrangement is one of the potential determinants of their level of LS.

**Methods:**

Data was drawn from the first and most recent wave of Longitudinal Ageing Study in India conducted in 2017–18. Using the Satisfaction with Life Scale, the level of LS was assessed for 30,370 elderly aged 60 + . Bivariate analysis was carried out to see the variation in the level of LS across elderlies with different socio-demographic characteristics. To investigate the association between LS and living arrangements and the selected socio-demographic factors multinomial logistic regression model was fitted.

**Result:**

The findings reveal that 25.4% and 45.5% of the elderlies have reported having a low and high level of LS, respectively. Living alone was associated with low level of LS. Co-residing with a spouse was associated with a higher likelihood of reporting high level of LS. The study also found that having both spouse and children as coresident increases the likelihood of reporting high level of LS (RRR = 3.15, 95%CI = 2.3–4.28). Elderly with self-reported poor health, limitation in more than two activities of daily living and presence of depressive symptoms were significantly associated with reporting low level of LS. However, being diagnosed with more than three chronic illnesses was associated with high level of LS (RRR = 1.41, 95%CI = 1.25–1.59). Older adults with the following characteristics were more likely to report a lower level of LS: male, 60–64 years old, no or few years of schooling, unmarried, working, rural resident, living in a poor household, Scheduled Caste and Tribe.

**Conclusion:**

The level of life satisfaction in Indian older adults is significantly associated with their living arrangements, thus suggesting that the LS of older adults could be facilitated through interventions that consider their living arrangements. Older adults with various personal and household characteristics were identified as vulnerable groups, who should be the prime targets of the existing welfare policies.

## Introduction

India has the world’s second-largest elderly population and one of the world’s fastest growth rates in the elderly population [1]. India would only take three more decades to double its 60 + population, which is projected to reach 289.5 million in 2050 from 139.6 million in 2020 [2]. The country’s growing elderly population poses a significant challenge in improving the older citizens’ quality of life. Because, with ageing, people face special challenges such as a reduction in physical function, cognition, personal autonomy and engagement in social activities [3, 4], making them dependent on others for their daily activities and requirements. Indian older adults predominantly reside with their immediate family members, and they receive the highest social support and care from them when in need [5, 6]. Nevertheless, the blend of urbanization and western culture and children relocating overseas due to rapid globalization has been affecting the family system and the living arrangements of older adults in India [1, 5, 7, 8]. As a result, the proportion of older adults co-residing with their children is declining sharply in India [2]; this has also led to fewer intergenerational households, which makes the provision of household-based social support more challenging [9]. Furthermore, the decline in fertility rate and increase in longevity are elevating the elderly dependency ratio in the country, which is estimated to increase from 15.9% in 2020 to 28.2% in 2050 [2]. With this exorbitant growth rate of the older adult population and an increase in the elderly dependency ratio, the change in the living arrangements poses a serious concern about the older adults’ overall well-being.

Henriques et al. [10] propose four domains of well-being in the nested model: subjective (happiness and LS); health and functioning (biological and psychological); environmental (both material and social environments); and values and ideology (the moral and ethical perspective of an external observer and evaluator). However, in the recent decade, policymakers and social scientists have been more inclined towards subjective measures of well-being to define and measure the welfare of individuals and the nation as a whole, because good health and economic conditions alone cannot make people happy and satisfy [11]. There is no doubt that no matter what personal and socio-economic characteristics one has, the main goal of the majority of people throughout the world is to be happy and satisfied with the life they lead [12]. Both happiness and LS are the subjective evaluation of one’s life; while happiness is affective feelings, LS is the cognitive evaluation [13]. Happiness is mostly based on emotional judgments, while LS is a cognitive assessment of the discrepancy between what you want and what you have; happiness is quite transitory, while LS tends to be stable (although it is sensitive to major events and changes in life conditions) [14]. Researchers consider LS as the measure of evaluated well-being because it requires the evaluation of one’s life events and experiences over relatively long periods [13, 15, 16]. Moreover, it is often considered as a common indicator of the overall well-being of a person [17], and a key index for measuring societal progress [18].

LS is more appealing to researchers assessing the quality of life of older adults because it is a more cognitive and stable phenomenon [11, 14]. For older adults, it is a multidimensional issue that is influenced by various objective life conditions, personal traits, and psychological characteristics [14, 19]. Previous studies in both developed and developing countries have identified a number of common predictors of LS in older adults, which include marital status, gender, employment, family ties, physical and mental health, social network, social support, wealth, participation in spiritual and religious events, education, social group, and caste in case of India [19–29]. In India, the existing studies on LS or similar concepts such as happiness and quality of life particularly focused on its association with socio-demographic factors [25, 30], social networks and support [26, 29, 31], change in living arrangements [32], household headship [33], and caste [34]. Yet the above studies have also found that living arrangement is a fundamental factor for LS of Indian older adults [20, 22, 25, 27, 29, 30, 32, 33]. However, none of these studies has extensively studied living arrangement as a significant predictor of LS except for Nagargoje et al. [29], who studied mediating effect of living arrangement in the relationship between social participation and life satisfaction.

A person’s living arrangement generally indicates with whom the person resides. For older adults, it can be alone, with a spouse, with their children, with a spouse and children, or with others [35]. And it cannot be ignored while discussing the LS of older adults in a country like India, where collectivist culture has remained as an important feature right from ancient times [6]. Studies indicate that the majority of Indian older adults co-reside with their immediate family members, and they have better health and well-being compared to those who live without their family [1, 4–6, 32, 36]. The most crucial aspect of living with family members is that kin provides financial, personal care and emotional support, help with day-to-day activities, and health awareness [4–6, 28], which have a significant impact on LS. But, alarmingly, a higher proportion of the perpetrators of crime against older adults are the immediate family members, relatives, neighbours, and caregivers [37]. Moreover, about 56.3% of the caregivers feel that the burden is mild to moderate, while 15.1% feel it is a severe burden to take care of dependent older adults [38]. Thus, these literatures may also indicate that older adults may not receive the same level of care and attention they had devoted to their ageing parents and parents-in-law. In these cases, dissatisfaction and sometimes even resentment seeps in when there is a gap between what one expects and what one gets.

In addition to the above discussion, other reasons that compelled the researchers of this study to investigate whether living arrangements is a predominant predictor of LS of Indian older adults are as follows: (i) living arrangement of older adults is significant in achieveing the sustainable development goal 3: “ensuring healthy lives and promoting well-being for all at all ages” [39]; (ii) the factors that were important in predicting LS of older adults in one country may not be relevant to their counterparts in another country. This is because even people in similar economic and non-economic circumstances may use different benchmarks or scales to evaluate their well-being, owing to differences in sociocultural norms and the relative value placed on various achievements [11]; and (iii) the paucity of research on living arrangement and LS of Older adults in the Indian context.

## Data and methods

### Data

**Fig. 1:**
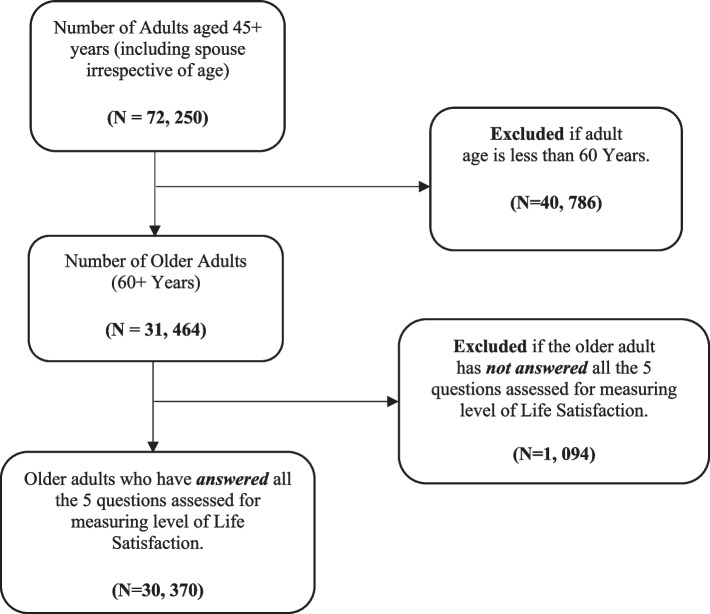
Sample selection flow chart

The present study has used the first and most recent wave of the Longitudinal Aging Survey of India (LASI), 2017–18 data. LASI has collected detailed information on healthcare practices, health behaviour and risk factors, healthcare utilization, and various demographic and socio-economic characteristics. In addition, information on older adults’ perceptions about their life, living arrangements and family relations was also collected from a 31,464 nationally representative adults population aged above 60 (referred to as older adults) in India [35]. Among those older adults, only 30,370 (Male-14,553; Female-15,817) responded to the questions about life satisfaction, and they were considered for the analysis in this study. The detailed sample selection procedure is presented in Fig. 1.

### Study variables

#### Dependent variable

In this study, the level of LS is considered as the main outcome variable. The Satisfaction with Life Scale (SWLS) was used to assess the level of LS among older adults. The scale was designed to assess an individual’s cognitive evaluation of overall LS [40]. The SWLS has good internal consistency and test–retest reliability [41] and is one of the most frequently used scales in subjective well-being research [13]. The SWLS is a very simple, short questionnaire made up of only five statements: “(i) In most ways, my life is close to ideal; (ii) the conditions of my life are excellent; (iii) I am satisfied with my life; (iv) so far, I have got the important things I want in life; and (v) if I could live my life again, I would change almost nothing”. A scale was assigned using the 7-point Likert scale (ranging from 1 = strongly disagree to 7 = strongly agree), indicating the respondents’ agreement with the five statements regarding LS. And an overall score was constructed by summing up the scales assigned for those five statements; therefore, the possible range of scores ranges from 5 to 35, with a score of 5–9, 10–14, 15–19, 20, 21–25, 26–30, and 31–35 indicating that the respondent is “Extremely Dissatisfied”, “Dissatisfied”, “Slightly Dissatisfied”, “Neutral”, “Slightly Satisfied”, “Satisfied”, and “Extremely Satisfied” with life, respectively [42]. However, for simplicity of computation and interpretation, we clubbed the seven levels of LS into three: (i) Low includes extremely dissatisfied, dissatisfied, and slightly dissatisfied; (ii) Medium includes neutral and slightly satisfied; and (iii) High includes satisfied and extremely satisfied. Hence, the scores for low, medium and high levels of LS are 5–19, 20–25, and 26–35, respectively.

#### Independent variable

Living arrangement was the independent variable in this study. We focused on conceptualizing it by building on earlier research [4, 5, 36, 39] and based on the categories as classified in LASI, 2017–18. The classification of living arrangements of older adults was of five mutually exclusive groups: (i) living alone – those living on their own; (ii) living with spouse only – those living only with their spouse; (iii) living with spouse and children – those living with their spouse and children; (iv) living with children only – those living with only children but not spouse: (v) living with others only – those living with the persons who are neither their spouse nor their children. It is to be noted that the other members (members excluding spouse and children) may also be co-residing in living arrangements (ii), (iii) and (iv); but in cases where an older adult who have co-residents but they are neither his/her spouse nor his/her children, the living arrangement is categorized as (v) living with others only.

#### Control variables

Aside from living arrangements, researchers have identified several variables that have been consistently linked with LS, including health, socio-economic status, education, and financial contentment [24, 43]. The individual characteristics selected for this study were sex (male and female), age group (60–64, 65–69, 70–74, and 75 +), years of schooling (no education, 1–4, 5–9, 10–14, and 15 +), marital status (married, widowed, and never married/divorced/separated/deserted), and working status (not working and working). The health indicators include self-rated health (moderate, good, and poor), limitation in activities of daily living (ADL), limitation in instrumental activities of daily living (IADL), number of chronic diseases (0, 1, 2, and 3 +) and depressive symptoms (Yes/No). Household characteristics include place of residence (rural and urban), social group (scheduled caste [SC], scheduled tribe [ST], other backward class [OBC] and Non-SC/ST/OBC), religion (Hindu, Muslim, Christian, and Others) economic status (middle, poor, and rich), and Region (North, West, Central, East, North-east, and South).

### Statistical method

Bivariate analysis was carried out to assess the distribution of Indian older adults by their levels of LS across the selected socio-demographic characteristics. Pearson’s chi-square test for independence was used to test whether or not there is a significant association between the levels of LS and the selected socio-demographic characteristics variables. To estimate the effects of living arrangements adjusted for a set of health, individual and household (HH) characteristics on the dependent variable, that is, the level of LS, a multinomial logistic (MNL) regression model was fitted using the “mlogit” function in Stata 14 software. Before considering the MNL regression model for the analysis, the proportional odds (PO) model was fitted considering the ordinal nature of the dependent variable; and the omnibus Brant test [44] of the PO assumption was conducted. The Brant test’s χ^2^ (9) = 28.68, *p* < 0.001, indicated that the PO assumption for the model was violated. And the assumption of the PO model also did not meet for any of the predictor variables. All the statistical analyses were carried out using the statistical software package Stata/SE, version 14.

The general MNL regression model for this study is specified according to Schmidt and Strauss[45]:

1 $$Pr\left({Y}_{i}=j\right)=\frac{{e}^{{\beta }_{j}{X}_{i}}}{1+\sum_{k=1}^{J}{e}^{{\beta }_{j}{X}_{i} }} ,\mathrm{ j}=1, 2, 3$$

where $Pr\left({Y}_{i}=j\right)$ denotes the probability of an individual *‘i’* being in the outcome category *‘j’*. *‘J’* is the number of categories in the outcome variables; in this case, it is 3, hence *‘j’* takes a value of 1, 2, or 3. ‘${\beta }_{j}$’ is the regression coefficient. Owing to the three categories of the dependent variable in the study, the model has two equations. The simplified equations are as follows:

2 $$\mathrm{ln}\left[\frac{\mathrm{Pr}(Y=Medium)}{\mathrm{Pr}(Y=Low)}\right]= {\alpha }_{k}^{m}x+{\beta }_{k}^{m}Y+{\delta }_{k}^{m}H+{\gamma }_{k}^{m}Z+{\varepsilon }_{k}^{m}$$

3 $$\mathrm{ln}\left[\frac{\mathrm{Pr}(Y=High)}{\mathrm{Pr}(Y=Low)}\right]= {\alpha }_{k}^{h}x+{\beta }_{k}^{h}Y+{\delta }_{k}^{h}H+{\gamma }_{k}^{h}Z+{\varepsilon }_{k}^{h}$$

Equation 2 represents the log odds (logit) of reporting medium level of LS relative to low level of LS. Similarly, Eq. 3 is for the log odds of reporting high level of LS relative to low level of LS. In both the equations, $x$ is the type of living arrangement, and $Y, H\mathrm{ and }Z$ are the sets of individual, health and household characteristics, respectively; and ${\alpha }_{k},{\beta }_{k}, {\delta }_{k},and {\gamma }_{k}$ are the respective slope coefficients, and ${\varepsilon }_{k}$ is the error term for the respective equations. In this study, the estimated parameters represent the RRR. An RRR > 1 indicates the likelihood of being in the comparison group (medium or high level of LS) relative to the likelihood of being in the referent group (low level of LS) for the comparison groups in the independent variable is higher than that of their referent group counterpart. In other words, the comparison group of the independent variable is more likely to report “medium/high level of LS”. Explained in another way, an RRR < 1 indicates that the likelihood of reporting “medium/high level of LS” relative to reporting “low level of LS” is lower for the comparison groups of the independent variable (e.g., living with a spouse, with spouse and children, and with others only) as compared to the referent group of the independent variable (e.g., living alone).

## Results

Table 1 presents the characteristics of the sample. More than two-fifths (43.5%) of the elderly were living ‘with their spouse and children’, more than one-fifth (26.7%) ‘with children and others’, and nearly one-fifth (19.5%) ‘with spouse and others’. About 52% are females, and 48% are males. More than half (53.4%) of the samples have not received formal education. One-third (33.7%) of the samples were widowed, and 64.3% were married. More than three-quarters (77%) of the sample reported moderate or good health, and about 46% had depressive symptoms. More than one-fifth (20.4%) of the older adults reported having at least one limitation in ADL, 43.5% reported having at least one limitation in IADL, and nearly one-fourth (24.6%) reported having at least two chronic diseases. Nearly two-thirds of the sample belongs to rural areas. OBC constitute 37.8%, while SC and ST share an almost equal proportion of the total sample. The majority of the sample were from Hindu households (73.4%), followed by Muslim (11.8%) and Christian (9.9%). Southern states have nearly one-fourth (24%) of the sample size.

**Table 1:** Mean life satisfaction score, and percentage distribution of older adults by their levels of life satisfaction by background characteristics, India, 2017–18

	**Mean Score of Life Satisfaction (SE)**	**Level of Life Satisfaction**			**Sample Size**
		**Low**	**Medium**	**High**	**N (%)**
**Current Living Arrangement**					
Living Alone	20.6 (0.21)	41.8	25.3	32.9	1563 (5.1)
With Spouse and/or Others	23.7 (0.1)	25.3	29.4	45.3	5918 (19.5)
With Spouse and Children	24.5 (0.06)	21.8	29.6	48.5	13,221 (43.5)
With Children and Others	23.7 (0.08)	25.6	29.1	45.3	8110 (26.7)
With Others Only	21.7 (0.21)	34.5	28.8	36.7	1558 (5.1)
**Individual Characteristics**					
**Sex**					
Male	24.1 (0.06)	24.0	28.8	47.2	14,553 (47.9)
Female	23.4 (0.06)	26.5	29.5	44.0	15,817 (52.1)
**Age Groups**					
60–64	23.8 (0.08)	25.3	30.1	44.6	9908 (32.6)
65–69	23.8 (0.08)	24.9	28.6	46.4	8617 (28.4)
70–74	23.7 (0.1)	25.0	28.9	46.1	5538 (18.2)
75 +	23.5 (0.1)	26.4	28.7	44.9	6307 (20.8)
**Years of Schooling**					
No Education	22.6 (0.06)	29.8	31.4	38.8	16,224 (53.4)
1–4 Years	23.8 (0.13)	25.6	29.2	45.1	3670 (12.1)
5–9 Years	24.8 (0.1)	20.3	27.8	51.9	5837 (19.2)
10 + Years	26.8 (0.12)	13.9	21.8	64.3	4639 (15.3)
**Marital Status**					
Married	24.2 (0.05)	23.3	29.5	47.3	19,527 (64.3)
Widowed	23.1 (0.08)	28.5	28.5	43.0	10,223 (33.7)
Never Married/Divorced/Separated/Deserted	21.6 (0.32)	35.8	31.1	33.1	620 (2)
**Working Status**					
Not Working	23.7 (0.05)	25.2	28.8	46.0	21,271 (70)
Working	23.7 (0.08)	25.8	29.9	44.4	9099 (30)
**Health Indicators**					
**Self-Rated Health**					
Moderate	23.9 (0.06)	23.8	30.7	45.5	13,194 (43.5)
Good	25.4 (0.07)	19.5	26.2	54.3	10,164 (33.5)
Poor	21.4 (0.1)	35.8	29.9	34.3	6996 (23)
**Limitation in ADL**					
No Limitation	24.1 (0.05)	24.4	28.6	47.1	24,158 (79.6)
1	23.3 (0.15)	27.1	27.8	45.1	2651 (8.7)
2 +	22.2 (0.14)	30.0	33.4	36.6	3556 (11.7)
**Limitation in IADL**					
No Limitation	24.5 (0.06)	22.6	28.5	48.9	17,147 (56.5)
1	24 (0.13)	25.4	27.6	46.9	3355 (11.1)
2 +	22.5 (0.08)	29.4	30.6	40.0	9824 (32.4)
**No. of Chronic Diseases**					
None	23.7 (0.06)	25.1	30.9	44.0	13,916 (45.9)
1	23.6 (0.08)	26.0	28.2	45.8	8953 (29.5)
2	23.5 (0.11)	25.7	29.7	44.6	4952 (16.3)
3 +	24.6 (0.15)	23.6	21.5	54.9	2530 (8.3)
**Depressive Symptoms**					
Not Depressed	25.7 (0.05)	16.6	26.4	57.0	16,414 (54.1)
Depressive Symptoms	21.6 (0.07)	34.8	32.2	33.0	13,901 (45.9)
**Household Characteristics**					
**Residence**					
Urban	25 (0.08)	21.2	25.1	53.7	10,334 (34)
Rural	23.2 (0.05)	27.1	30.8	42.2	20,036 (66)
**Social Group**					
Non-SC/ST/OBC	24.9 (0.08)	20.1	29.0	50.9	8944 (29.5)
SC	22.5 (0.11)	31.0	31.5	37.5	4965 (16.3)
ST	22.9 (0.1)	29.5	30.7	39.8	4970 (16.4)
OBC	23.7 (0.07)	25.5	27.9	46.6	11,491 (37.8)
**Religion**					
Hindu	23.7 (0.05)	25.5	28.9	45.6	22,283 (73.4)
Muslim	23.8 (0.12)	24.9	31.7	43.5	3582 (11.8)
Christian	22.9 (0.15)	29.4	26.1	44.5	3009 (9.9)
Others	24.5 (0.19)	20.8	29.5	49.7	1496 (4.9)
**Economic Status**					
Middle	24.1 (0.09)	23.8	29.3	46.9	6203 (20.4)
Poor	23 (0.07)	28.1	31.6	40.3	12,469 (41.1)
Rich	24.4 (0.07)	23.0	26.0	51.0	11,698 (38.5)
**Region**					
North	23.6 (0.09)	25.2	32.9	41.9	5657 (18.6)
West	27.2 (0.11)	12.2	19.2	68.6	4133 (13.6)
Central	23.5 (0.12)	25.0	33.2	41.8	4086 (13.5)
East	23.1 (0.09)	27.0	35.3	37.6	5601 (18.4)
North-east	24.2 (0.1)	20.2	34.6	45.2	3602 (11.9)
South	22 (0.1)	34.9	23.5	41.6	7291 (24)
**India**	**23.7 (0.04)**	**25.4**	**29.1**	**45.5**	**30,370**

**Fig. 2:**
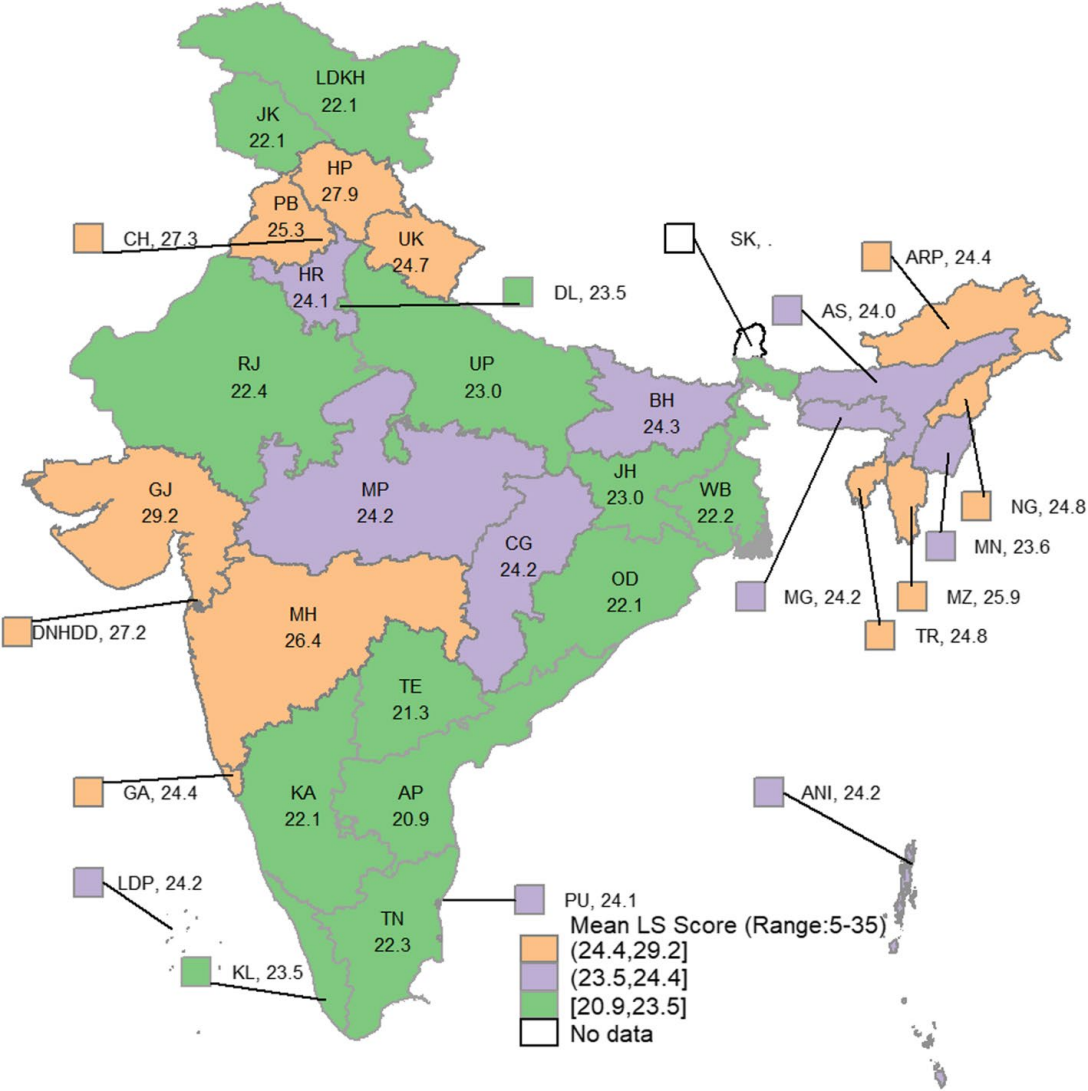
Mean LS score of the older adults across the States and union territories of India, 2017–18 Note: The Map was developed by the authors using Stata software version 14.1, and the map was cross verified with the India map and its States and Union Territories’ boundaries as shown in the official website of Survey of India: https://indiamaps.gov.in/soiapp/. State and Union Territories: ANI: Andaman & Nicobar Islands; AP: Andhra Pradesh; ARP: Arunachal Pradesh; AS: Assam; BH: Bihar; CG: Chhattisgarh; CH: Chandigarh; DL: Delhi; DNHDD: Dadra & Nagar Haveli, and Daman & Diu; GA:Goa; GJ: Gujarat; HP: Himachal Pradesh; HR: Haryana; JH: Jharkhand; JK: Jammu & Kashmir; KA: Karnataka; KL: Kerala; LDKH: Ladakh; LDP: Lakshadweep; MG: Meghalaya; MH: Maharashtra; MN: Manipur; MP: Madhya Pradesh; MZ: Mizoram; NG: Nagaland; OD: Odisha; PB: Punjab; PU: Puducherry; RJ: Rajasthan; SK: Sikkim; TE: Telangana; TN: Tamil Nadu; TR: Tripura; UK: Uttarakhand; UP: Uttar Pradesh; WB: West Bengal

**Fig. 3:**
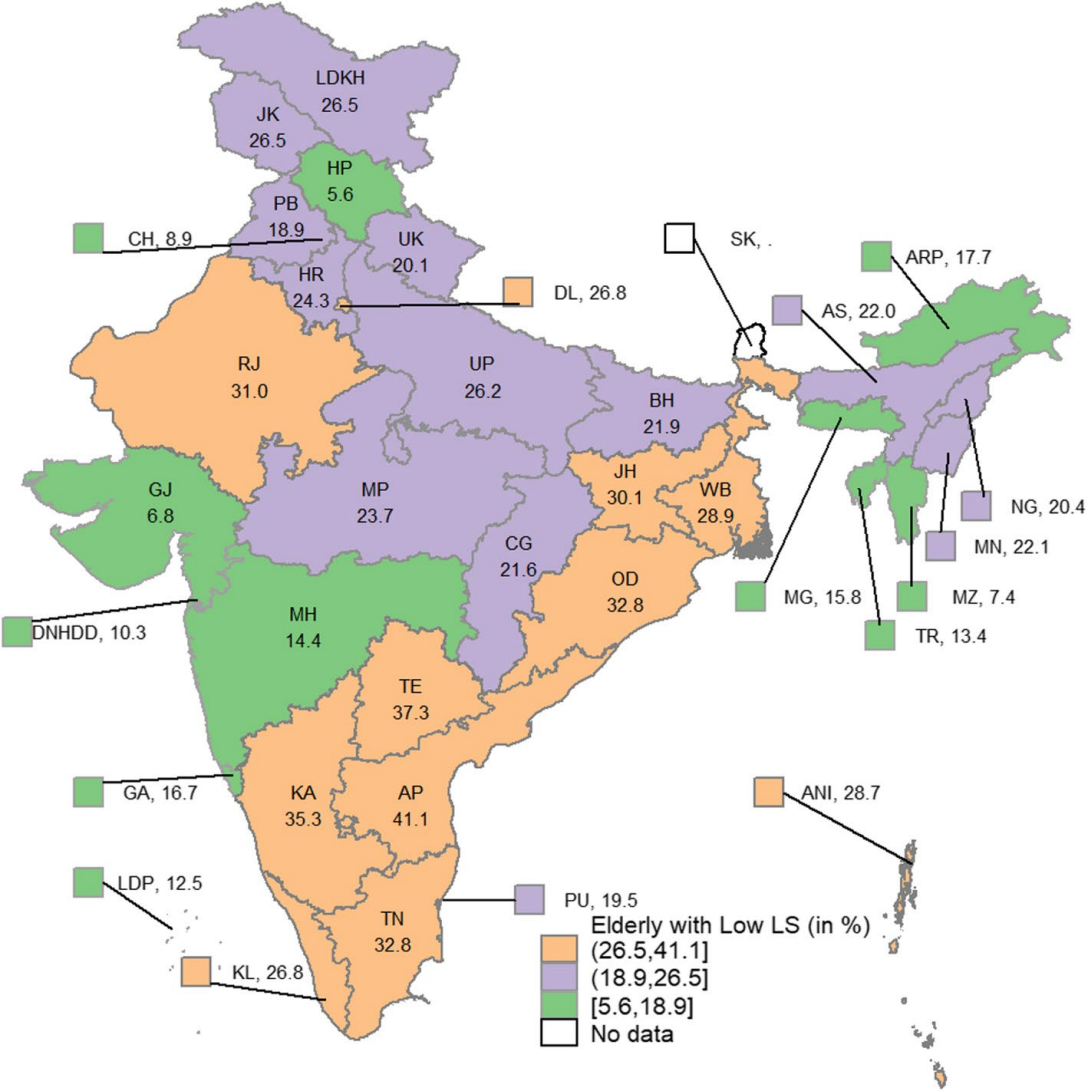
Percentage of older adults with low level of LS across the States and union territories of India, 2017–18 Note: State and union territories: Same as in Fig. 2

Table 1 also presents the mean LS score across the sub-populations and the distribution of that sub-population by their LS levels. One-fourth (25.4%) of the 60 + population reported a lower level of LS, while 45.5% reported a high level of LS. From Table 1, it is also clear that noticeable differences exist in all five household characteristics and in some personal traits such as years of schooling, marital status, self-rated health, limitation in ADL and IADL, number of chronic diseases, and depressive symptoms. In household characteristics, older adults from rural, SC, ST, Christian, and economically poorer households have reported low level of LS at a greater rate than their other counterparts. Among the regions of India, one in every three (34.9%) older adults in the southern region have reported a low level of LS, which is remarkably higher than in other regions. To help visualize more clearly how LS varies across the states and union territories of the country, mean LS Scores and the percentage of older adults who reported having low levels of LS for the states and union territories of India are provided in Figs. 2 and 3, respectively.

**Fig. 4:**
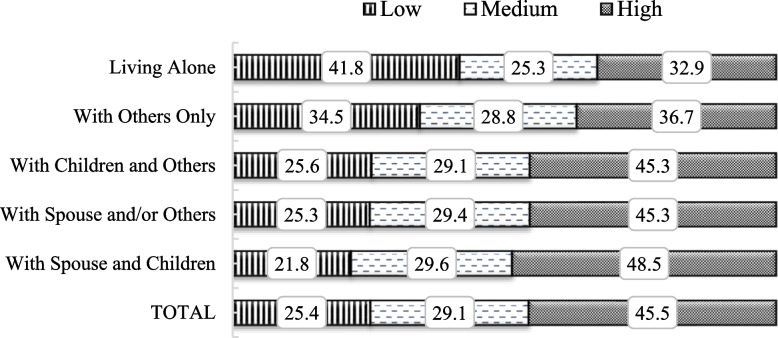
Percentage distribution of older adults by their levels of LS across the living arrangements, India, 2017–18

Figure 4 shows the percentage distribution of older adults based on their levels of LS in different living arrangements. Compared to the other living arrangements, older adults who were living alone reported low level of LS at a more significant rate (41.8%), and older adults who were living with their spouse and children reported high level of LS at a larger rate (45.5%). Overall, a remarkably higher proportion of older adults who do not live with their spouse or children have reported low level of LS.

Results from the MNL regression model of LS are presented in Table 2. The results are presented in terms of relative risk ratio (RRR) and its 95% confidence interval (CI). A RRR value of greater than 1 indicates that older adults from that category are more likely to report higher level of LS when compared with the elderly in the respective reference category. For instance, we found elderly co-residing ‘with both spouse and children’ were significantly more likely to report a higher level of LS (RRR for Medium = 2.46 & High = 3.15, *p*-value < 0.001), followed by those who are living ‘with their spouse only’ (RRR for Medium = 2.34 & High = 2.76, *p*-value < 0.001). Though compared to the older adults who were living alone, the RRR of reporting medium and high levels of LS is greater for elderly living ‘with their children only’, it is remarkably lower than the RRR estimated for ‘living with Spouse’ and ‘living with spouse and children’ living arrangements. The result also revealed that older adults who are living alone are significantly more likely to report lower levels of LS compared to the elderly in other living arrangements.

**Table 2:** Multinomial logistic regression result for the association of levels of life satisfaction in older adults with their living arrangements and other background characteristics

	**Model-1**		**Model-2**		**Model-3**		**Model-4**	
	**Medium**	**High**	**Medium**	**High**	**Medium**	**High**	**Medium**	**High**
**Living Arrangement** (Ref: Living Alone)								
With Spouse	1.927*** [1.68,2.21]	2.275*** [2.00,2.58]	3.113*** [2.27,4.26]	3.576*** [2.68,4.78]	2.810*** [2.04,3.86]	2.975*** [2.20,4.03]	2.344*** [1.70,3.24]	2.764*** [2.03,3.77]
With Spouse and Children	2.249*** [1.98,2.56]	2.823*** [2.51,3.18]	3.608*** [2.64,4.93]	4.299*** [3.23,5.73]	3.186*** [2.33,4.36]	3.430*** [2.54,4.63]	2.463*** [1.79,3.39]	3.147*** [2.31,4.28]
With Children and Others	1.883*** [1.65,2.15]	2.251*** [1.99,2.54]	1.879*** [1.64,2.15]	2.146*** [1.90,2.43]	1.731*** [1.51,1.98]	1.804*** [1.58,2.05]	1.606*** [1.40,1.84]	1.833*** [1.60,2.09]
With Others Only	1.378*** [1.16,1.64]	1.348*** [1.15,1.58]	1.432*** [1.20,1.70]	1.447*** [1.23,1.70]	1.379*** [1.16,1.64]	1.345*** [1.13,1.59]	1.299** [1.09,1.55]	1.422*** [1.19,1.69]
**Individual Characteristics**								
**Sex** (Ref: Male)								
Female			1.111** [1.03,1.20]	1.279*** [1.19,1.37]	1.122** [1.04,1.21]	1.274*** [1.19,1.37]	1.121** [1.04,1.21]	1.180*** [1.10,1.27]
**Age Groups** (Ref: 60–64 Years)								
65–69			1.019 [0.94,1.10]	1.146*** [1.06,1.24]	1.013 [0.93,1.10]	1.125** [1.04,1.22]	1.04 [0.96,1.13]	1.117** [1.03,1.21]
70–74			1.074 [0.98,1.18]	1.206*** [1.11,1.32]	1.065 [0.97,1.17]	1.173*** [1.07,1.28]	1.064 [0.97,1.17]	1.142** [1.04,1.25]
75 +			1.043 [0.95,1.14]	1.217*** [1.12,1.33]	1.07 [0.97,1.18]	1.342*** [1.23,1.47]	1.061 [0.96,1.17]	1.325*** [1.21,1.45]
**Years of Schooling** (Ref: No Education)								
1–4 Years			1.073 [0.97,1.19]	1.390*** [1.27,1.52]	1.069 [0.97,1.18]	1.299*** [1.18,1.43]	1.057 [0.95,1.17]	1.089 [0.99,1.20]
5–9 Years			1.275*** [1.17,1.39]	2.019*** [1.86,2.19]	1.285*** [1.17,1.41]	1.890*** [1.73,2.06]	1.282*** [1.17,1.41]	1.723*** [1.58,1.88]
10 + Years			1.449*** [1.29,1.63]	3.700*** [3.34,4.09]	1.407*** [1.25,1.58]	2.997*** [2.69,3.33]	1.362*** [1.20,1.54]	2.471*** [2.21,2.76]
**Marital Status** (Ref: Married)								
Widowed			1.622** [1.21,2.17]	1.698*** [1.30,2.22]	1.576** [1.18,2.11]	1.693*** [1.28,2.24]	1.386* [1.03,1.87]	1.579** [1.19,2.10]
Never Married/Divorced/Separated/Deserted			1.633** [1.15,2.33]	1.213 [0.87,1.70]	1.562* [1.09,2.23]	1.227 [0.86,1.74]	1.363 [0.95,1.96]	1.055 [0.74,1.51]
**Working Status** (Ref: Not Working)								
Working			1.023 [0.95,1.10]	1.028 [0.96,1.10]	0.959 [0.89,1.03]	0.933 [0.87,1.00]	0.983 [0.91,1.06]	0.901** [0.84,0.97]
**Health Indicators**								
**Self-Rated Health** (Ref: Moderate)								
Good					0.978 [0.90,1.06]	1.310*** [1.22,1.41]	0.979 [0.90,1.06]	1.278*** [1.19,1.38]
Poor					0.701*** [0.65,0.76]	0.577*** [0.54,0.62]	0.729*** [0.67,0.79]	0.638*** [0.59,0.69]
**Limitation in ADL** (Ref: No Limitation)								
1					0.978 [0.88,1.09]	1.061 [0.96,1.17]	0.929 [0.83,1.04]	0.929 [0.84,1.03]
2 +					1.188*** [1.08,1.30]	0.948 [0.86,1.04]	1.131* [1.03,1.24]	0.787*** [0.71,0.87]
**No. of Chronic Diseases** (Ref: None)								
1					0.897** [0.83,0.97]	1.036 [0.96,1.11]	0.93 [0.86,1.00]	0.998 [0.93,1.07]
2					0.976 [0.89,1.07]	1.054 [0.96,1.15]	1.038 [0.94,1.14]	0.995 [0.91,1.09]
3 +					0.767*** [0.67,0.88]	1.434*** [1.27,1.61]	0.852* [0.74,0.98]	1.410*** [1.25,1.59]
**Depressive Symptoms** (Ref: No)								
Yes					0.627*** [0.59,0.67]	0.324*** [0.30,0.34]	0.628*** [0.59,0.67]	0.340*** [0.32,0.36]
**Household Characteristics**								
**Social Groups** (Ref: Non-SC/ST/OBC)								
SC							0.836*** [0.76,0.92]	0.756*** [0.69,0.83]
ST							0.737*** [0.65,0.84]	0.656*** [0.58,0.75]
OBC							0.961 [0.88,1.05]	1.016 [0.94,1.10]
**Religion** (Ref: Hindu)								
Muslim							1.011 [0.91,1.12]	0.926 [0.84,1.03]
Christian							1.073 [0.88,1.30]	1.306** [1.09,1.57]
Others							1.081 [0.90,1.30]	1.03 [0.86,1.23]
**Economic Status** (Ref: Middle)								
Poor							0.909* [0.84,0.99]	0.773*** [0.71,0.84]
Rich							0.931 [0.85,1.02]	1.06 [0.97,1.15]
**Region** (Ref: North)								
West							1.186* [1.04,1.36]	3.493*** [3.08,3.96]
Central							1.120* [1.00,1.25]	1.320*** [1.18,1.47]
East							1.045 [0.94,1.16]	0.986 [0.88,1.10]
North-east							1.297* [1.05,1.60]	1.292* [1.05,1.59]
South							0.563*** [0.50,0.63]	0.777*** [0.69,0.87]

The result is presented in terms of Relative Risk Ratio (RRR); 95% confidence intervals of RRRs are in brackets ‘[]’; Ref: indicates the reference category of the variable

Significance Level: * *p* < 0.05, ** *p* < 0.01, *** *p* < 0.001

After applying the backward elimination method to eliminate the variables that did not show any statistically significant association, the final model (model-4) retained individual characteristics, health indicators, and household characteristics that are found to be significant determinants of LS, which are presented in Table 2. The RRR revealed that female older adults are statistically significantly more likely to report a higher level of LS (RRR for Medium = 1.12 & High = 1.18, *p*-value < 0.001). Older adults who were older in age and who had higher schooling years were more likely to report higher levels of LS compared to their younger and lower schooling years counterparts. It is quite astonishing to note that widowed older adults are more likely to report medium or high levels of LS compared to married older adults (RRR for Medium = 1.39 & High = 1.58, *p*-value < 0.001). Working older adults are 10% less likely to report high level of LS (RRR for High = 0.9, *p*-value < 0.001).

Similarly, in health indicators, self-reported poor health (RRR for Medium = 0.73 & High = 0.64, *p*-value < 0.001), limitation in more than two ADLs (RRR for High = 0.79, *p*-value < 0.001), and presence of depressive symptoms (RRR for Medium = 0.63 & High = 0.34, *p*-value < 0.001) were significantly associated with reporting a lower level of LS. It is quite astonishing to note that older adults diagnosed with 3 or more chronic conditions were significantly more likely to report high level of LS (RRR for High = 1.41, *p*-value < 0.001), but less likely to report medium level of LS (RRR for High = 0.85, *p*-value < 0.001).

In HH characteristics, elderly from ST HH (RRR for Medium = 0.73 & High = 0.66, *p*-value < 0.001), SC HH (RRR for Medium = 0.84 & High = 0.76, *p*-value < 0.001), economically poor HH (RRR for Medium = 0.91 & High = 0.77, *p*-value < 0.05), and from southern region (RRR for Medium = 0.56 & High = 0.78, *p*-value < 0.001), were less likely to report medium or high level of LS. Elderly living in HH practising Christianity (RRR for High = 1.31, *p*-value < 0.01) and living in Western, Central and North-eastern regions were more likely to report high level of LS.

## Discussion

This study examined the association between living arrangements and the level of LS in Indian older adults aged 60 + , adjusting for the selected health indicators, individual characteristics, and HH characteristics. The findings suggest that the level of LS of the elderly is statistically significantly associated with their living arrangements. “Living alone” is significantly associated with reporting low level of LS, followed by “living with others only”. In agreement with Kooshiar et al. [20], this study indicates that living in the same arrangement with a spouse is associated with reporting a higher level of satisfaction. Possible explanations for these findings can be as follows: loneliness can adversely affect the LS of an individual [20, 46]; and having spouse under the same roof provides a significant support system for the older adult —males can look after the finances and females can take care of health issues— [21]. Further, the study also found that having both the spouse and children as co-residents increases the likelihood of reporting a higher level of LS. This may be attributed to the presence of both spouse and children giving a feeling of having a complete family; and adult children usually shoulder their parents’ physical, mental and financial burden.

Concurrent with Singh & Singh [26], this study found no significant differences in the mean score of LS by the age and gender of the older adults. However, our study suggests female older adults were more likely to report higher levels of LS than males, and this finding is supported by Ng et al. [23]. Like the finding of Ng et al. [23], this study found an increase in the years of schooling is associated with an increase in mean LS score and a higher likelihood of reporting a higher level of LS, indicating older adults with “no” or “very few years” of schooling were more likely to report low level of LS. The possible explanation for this is that, post-retirement, an illiterate person, who was an unskilled worker, is less likely to receive a pension amount that can meet the exorbitant healthcare expenditures of an ill health older adults and the daily living expenditure of older adults in good health. This indicates financial insecurity may be higher among the illiterate and less educated elderly, due to which they may also be dissatisfied with the fact that they were once taking full responsibility for the upkeep of the house, but at present, instead of contributing to household expenses, they depend on the younger members. This study suggested low mean LS scores among the widowed and divorced/separated/deserted older adults. This may be because a negative event like the death of a spouse [47] and marital disruption causes a sharp decline in LS among older adults. Moreover, discrimination and negative labelling of people with a disrupted marriage are quite prevalent in Indian society, making them more vulnerable in their older ages, and to a significant extent, this affects their level of LS. Contrary to a previous study in India [29], our study found widow/widower are very more likely to report a higher level of LS compared to their married counterparts. This may be explained partially by the fact that widow shares a significantly higher proportion against the widowers in older adults with widowhood status; in India, widows may maintain economic independence by carrying on her spouse’s business and be accorded certain rights, such as entering guilds; and they may experience peace, and increase in their social participation and social support through attending religious and spiritual events. However, more robust research is required to determine the cause of such surprising findings.

The rate of reporting low level of LS is higher in older adults with more than two limitations in ADL or IADL, depressive symptoms and poor health. In line with previous studies [22, 25], the MNL regression result showed that having limitations in ADL was significantly associated with reporting low level of LS, but no association between IADL and the level of LS was established. In contrast to our study, Hsu & Jones [48] found no significant association between physical function difficulties (ADL and IADL) and LS. Unlike the previous study [22], our study indicates that having more than three chronic diseases is associated with a higher likelihood of reporting a high level of LS. To determine the reason for such unexpected finding, more robust research is required. Concurrent with Ng et al. [23] and Lombardo et al. [18], this study indicates that reporting good health is associated with reporting high level of LS, and reporting poor health is significantly linked to low level of LS. Poor physical health was hardly related to lower life satisfaction, whereas poor mental health was strongly related to lower life satisfaction.

In line with Muhammad et al. [34], this study found that living in a rural setting, belonging to SC and ST communities, and living in poor households is significantly linked to low level of LS in older adults. This may be ascribed to the likelihood of living alone is higher among these older adults compared to their counterparts [36]. It is well documented that illiteracy is higher among the rural and SC/ST population, and a higher proportion of SC/ST lives in rural areas; not to mention living in rural areas or from a poor household abstains older adults from availing better healthcare provisions and other social welfare services meant for the elderly. Several previous studies have also suggested that older adults from economically poorer households are significantly more likely to report low level of LS as compared to their economically richer counterparts [18, 22, 23, 48].

This study has the following limitations. First, it fails to draw any causal relationship between the LS and living arrangements due to cross-sectional nature of the dataset; hence, a longitudinal research design would be more precise to measure phenomena that changed over time. Second, while this study analyzed the association between living arrangement and LS, it did not investigate the factors that can influence living arrangements. Third, level of LS is assumed as the overall well-being of the elderly in this study; however, several factors, such as the mood or the surrounding environment when the interview was taken, in some cases influence the responses to items used for measuring the level of LS [49]. Further, LS is multidimensional; the perceived level of LS may vary from person to person based on individual expectations and requirements; moreover, recent events can also significantly impact evaluating life as a whole.

## Conclusion

The findings of this study showed that living with a spouse and children was the most common type of living arrangement for older adults in India as in other Asian countries. In India, co-residing with a spouse and living specifically along with a child was associated with a higher level of LS, and living alone was associated with a lower level of LS. The study also revealed that living arrangements could be assumed to be a crucial predictor of the LS of older adults in India. Significant socio-demographic disparity persists in the form of older adults’ satisfaction with their lives. Older adults with few years of schooling, with a disrupted marriage, from rural areas, belonging to poor households, SC and ST communities, and southern regions have reported low satisfaction with their lives.

The study identified various older adult groups with a higher likelihood of having a low level of LS. Thus, the existing welfare policies for older adults need to be redesigned to address the issues that hinder enhancing the satisfaction levels among these older adults. Early study indicates that the economic independence of the older population is a crucial indicator of their well-being, but nearly three-fourths of the older adults are economically dependent [1]; the Government needs to focus on financial incentives to ensure economic security. The number of older adults living alone is also increasing significantly, and they are more likely to have a low level of LS. Hence, there is a need to promote co-residence through policies, special care and provision to provide an enabling environment and to track the overall well-being of older adults living alone or at risk of becoming alone in the near future, such as older couples staying alone.

In a country where the population aged 60 years and over is growing faster than all younger age groups, it is important to continue exploring the scope of the problem by doing more robust research to address the issue of low LS and factors affecting older adults’ LS level. The study did not investigate any causal relationship, but this concern can be investigated with longitudinal studies, which is more appropriate for testing causality. Segregating “living with spouse”, “living with children”, and “living with spouse and children” that consider dependency of children (living with dependent children or independent children) and the health status of the spouse can give a more clear picture of the positive association of these two arrangements with the level of LS. Further research is warranted to see the factors that elevate the association between different living arrangements and the level of LS. This may help to enhance the existing welfare strategies to promote life satisfaction in older adults across all living arrangements.

## Data Availability

The study uses secondary data which is available in the public repository of International Institute for Population Sciences, Mumbai, which can be accessed by submitting the Data Request Form for Longitudinal Ageing Study in India (LASI), wave – I, 2017–18 available at: https://www.iipsindia.ac.in/content/LASI-data

## Abbreviations

LS: Life satisfaction
LASI: Longitudinal Ageing Study in India
SWLS: Satisfaction with Life Scale
HH: Household
ADL: Activities of Daily Living
IADL: Instrumental Activities of Daily Living
SC: Scheduled Caste
ST: Scheduled Tribe
OBC: Other Backward Class
MNL: Multinomial logistic
PO: Proportional Odds
RRR: Relative Risk Ratio
CI: Confidence Interval
